# The inverse relationship between fatness and bone mineral content is mediated by the adolescent appendicular skeletal muscle mass index: The Cogni-Action Project

**DOI:** 10.3389/fnut.2022.1040116

**Published:** 2022-11-15

**Authors:** Carlos Cristi-Montero, Humberto Peña-Jorquera, Leslie Landaeta-Díaz, Julio B. Mello, Felipe Araya-Quintanilla, Caroline Brand, Cézane Reuter, Carlos Jorquera, Gerson Ferrari

**Affiliations:** ^1^IRyS Group, Physical Education School, Pontificia Universidad Católica de Valparaíso, Valparaíso, Chile; ^2^Facultad de Salud y Ciencias Sociales, Universidad de las Américas, Santiago, Chile; ^3^Physical Education School, Faculdade SOGIPA, Porto Alegre, Brazil; ^4^eFiDac Group, Physical Education School, Pontificia Universidad Católica de Valparaíso, Valparaíso, Chile; ^5^Escuela de Kinesiología, Facultad de Odontología y Ciencias de la Rehabilitación, Universidad San Sebastián, Santiago, Chile; ^6^Graduate Program in Health Promotion, Universidade de Santa Cruz do Sul—UNISC, Santa Cruz do Sul, Brazil; ^7^Escuela de Nutrición y Dietética, Facultad de Ciencias, Universidad Mayor, Santiago, Chile; ^8^Escuela de Ciencias de la Actividad Física, el Deporte y la Salud, Universidad de Santiago de Chile (USACH), Santiago, Chile

**Keywords:** children, obesity, bone health, skeletal muscle mass index, physical activity

## Abstract

**Background:**

Excess adipose tissue negatively influences bone health during childhood, affecting future bone fragility diseases such as osteoporosis. However, little is known about how adolescent appendicular skeletal muscle mass index (ASMI) may mediate the relation between fatness and bone mineral content (BMC).

**Methods:**

The sample comprised 1,296 adolescents (50% girls) aged 10–14. A principal component analysis was performed to obtain a factor made up of four fatness indicators (a) neck circumference, (b) kilograms of fat, (c) visceral fat area, and (d) waist-to-height ratio. BMC, kilograms of fat, visceral fat area, and appendicular skeletal muscle mass were obtained by a multi-frequency bioelectrical impedance analyzer. ASMI was calculated as the appendicular skeletal muscle mass divided by height squared (kg/m^2^). A mediation analysis was performed adjusting by age, sex, maturation, socioeconomic status, physical activity, and adolescents' body weight. We also explore differences by sex and nutritional status.

**Results:**

The fatness factor explained 71.5% of the proportion variance. Fatness was inversely associated with the ASMI and BMC, while the ASMI was positively related to BMC. Overall, the inverse relationship between fatness and BMC was partially mediated by the adolescents' ASMI (29.7%, indirect effect: B= −0.048, 95%CI −0.077 to −0.022), being higher in girls than in boys (32.9 vs. 29.2%). Besides, the mediation effect was higher in adolescents with normal body weight than with overweight-obese (37.6 vs 23.9%, respectively).

**Conclusions:**

This finding highlighted the relevance of promoting healthy habits to reduce fatness and improve muscle mass in adolescents. Moreover, this highlights the central role of ASMI mediating the inverse association between fatness and BMC in female and male adolescents. Public health strategies should promote bone health in childhood, reducing the incidence of early osteopenia and osteoporosis.

## Introduction

A significant upward trend in the prevalence of overweight and obesity prevalence in adolescents worldwide has recently been reported (1, 2). Several fatness indicators [i.e., neck circumference, body mass index, waist-to-height ratio (WHtR), fat mass, visceral fat, and others] lead to various health concerns, but they have mainly been associated with an increased risk of chronic non-communicable diseases. However, little is known about the impact on adolescents' bone health. In fact, in 2020, a World Health Organization panel of experts agreed that bone health is a critical outcome in children and adolescents that needs to be studied in greater depth (3).

Adolescence is a crucial stage for forming a solid skeleton because the bones grow in size and strength (4, 5). The acquired bone mass in this stage (especially in the years around peak height velocity) represents approximately double the amount of bone mineral that subsequently will be lost between the ages of 50–80 years (6), and it can prevent future diseases of bone fragility such as osteopenia and osteoporosis (7, 8). Unfortunately, excess adiposity is unfavorable to bone health in childhood due to the cross-talk between adipose tissue and bone, which regulate each other reciprocally (9). For instance, adipocytes are active cells that can release pro-inflammatory and immunomodulatory molecules through endocrine and paracrine responses (10–12). This action promotes differentiation from multipotent mesenchymal stem cells into adipocytes instead of osteoblast and can up-regulate osteoclast formation and activation (10–12). This dysregulation between osteoblast/osteoclast cells due to excess adiposity favoring bone frailty is a plausible reason obese children show a higher rate of extremity fractures (13), including low vitamin D levels due to sequestration by an excess of fat tissue (14), even though body weight is a mechanical load favorable for bone accrual (15).

In this regard, muscular tissue arises as a relevant agent to controlling excess adiposity and improving bone health (16), being levels of physical activity (especially strength and high-impact activities), a crucial precursor positively associated with muscle mass and increased bone mineralization in adolescents (17, 18). Thus, ASMI (i.e., appendicular skeletal muscle mass adjusted by height squared) emerges as an interesting indicator to explore the interplay between fatness and bone health. This interaction, which has been related to future osteoporosis (19), seems to be a more sensitive biomarker of disease than body mass index (20), and the use of a value adjusted to the height is more appropriate than using the percentage of lean mass due to the possible mask of gains in lean mass during growth (21). However, the ASMI has been used broadly in older adults (22, 23) but not in adolescents. One of the few studies in adolescents found a positive relationship between ASMI and bone mineral content (BMC), and this relationship remains significant even after adjusting for fat mass (24). Nevertheless, the mediation role of ASMI on the relationship between fatness and BMC in this population is unknown.

This finding could be relevant considering the high prevalence of obesity, the low level of muscular fitness (25, 26), and sarcopenia in children and adolescents (27), which may drastically increase the future prevalence of osteoporosis (28). Therefore, this study seeks to evaluate the mediation role of ASMI in the relationship between fatness and BMC in a large sample of Chilean adolescents.

## Materials and methods

### Study design

This cross-sectional study is part of the Cogni-Action Project, which was approved by the Bioethics Committee of the Pontificia Universidad Católica de Valparaíso (BIOEPUCV-H103-2016). More details about the design and methodology of this project can be found elsewhere (29). The school principal, parents, and participants provided written informed consent before data collection. All aspects of the study were in accordance with the Declaration of Helsinki. The present study was performed according to STROBE guidelines (Strengthening the Reporting of Observational Studies in Epidemiology) for cross-sectional studies (30).

### Study population

A total of 1,296 adolescents (11.9 ± 1.1 years old) from 19 public, subsidized, and private schools participated in this study (50% girls and 50% boys). Total sample size and power calculations were based on the total enrollment of schoolchildren in the Valparaiso region (5th to 8th grades) indicated by the Chilean Ministry of Education in the year 2016 (universe *N* = 951 962). An alpha error of 5%, confidence interval of 99%, heterogeneity of 50%, and a 20% dropout were considered. Hence, a minimum of 797 participants were necessary to reach a representative sample size from the second most populated region in Chile. Exclusion criteria were: (a) being out of the stipulated age range, (b) missed the cognitive evaluation, or (c) not having data on any of the variables involved for this study. More details can be found elsewhere (29).

### Measurements

All measurements took place at schools between 9:00 and 15:00, in two sessions of 4 h each, separated by 8 days. Anthropometric and bioelectrical impedance analysis (BIA) evaluations were organized to minimize the influence of body weight variation due to sports and the consumption of liquids and foods. Adolescents were never assessed after lunch, maintaining a standardized protocol for the evaluation. We divided the classroom into two sections, one for anthropometric measures (under a restricted visual space) and the second for the BIA. All measurements were performed by previously trained members of our research group. Adolescents were asked to wear shorts, t-shirts, and no socks during all measurements. A teacher from the school always was present to safeguard all procedures.

### Anthropometric measures

The weight was measured with a balance (OMROM, HN-289-LA, Kyoto, Japan), with a precision of 0.1 kg, while the height, with a stadiometer (SECA, model 213, mbH, Germany) with a precision of 0.1 cm. Adolescent body mass index (BMI) was calculated from measurements of height and weight as a measure of adiposity (weight in kilograms divided by the square of height in meters). Cutoffs for defining normal, overweight, and obesity where based on the IOTF classification (31), which were then dichotomized (normal-BMI and overweight-obesity). Waist circumference (cm) was measured with an inextensible tape measure (Lufkin, Apex, NC) on a horizontal plane at minimal waist level at the end of a normal expiration. Neck circumference (cm) was measured with the subject standing in the most prominent portion of the stipulated zone with an inextensible tape measure (Lufkin, Apex, NC, USA) with the head placed in the Frankfort plane. Neck circumference has been associated with several fatness indicators and diagnosis of obesity, and in turn, with cardiometabolic risk in children and adolescents (32, 33). In a sample of Brazilian adolescents, neck circumference was around 32.6 ± 2.8 cm (33). WHtR was calculated by dividing the waist circumference by height, both in centimeters. WHtR is a close indicator of visceral or central fatness (intra-abdominal) being strongly related to development of cardiovascular disease and type 2 diabetes in adulthood (34). In a sample of Chilean children and adolescents, WHtR was around 0.47 to 0.49 (35).

### Bioelectrical impedance analysis

Participants were seated for 3–5 min in the resting position before beginning the bioelectrical impedance analysis (BIA) (arms abducted by 30° and the legs separated by 45° approx.). Meanwhile, eight electrodes were placed on the body, two on each hand and two on each ankle. The examiner prepared the skin with a clean alcohol wipe (70%) and attached electrodes. The analysis lasted around 2 mins for each adolescent.

The InBody S10 multi-frequency BIA analyzer (Biospace, Seoul, Korea) was used to measure the visceral fat area (VFA, cm^2^), total fat mass (kg), BMC (kg), and appendicular skeletal muscle mass (kg). The InBody S10 is a quick assessment that analyzes body composition in five segments of the body (i.e., arms, trunk, and legs) at six different frequencies (1, 5, 50, 250, 500, and 1,000 kHz) and has a strong correlation with the *gold standard* method (dual-energy X-ray absorptiometry) (36, 37).

### Appendicular Skeletal Muscle Mass Index

The Appendicular Skeletal Muscle Mass Index (ASMI) was calculated as the appendicular skeletal muscle mass of the limbs obtained by BIA divided by height squared (kg/m^2^) (24).

### Covariates

The primary mediation analysis (boys and girls together) was adjusted for sex, maturation, school vulnerability (i.e., proxy of socioeconomic indicator), and adolescents' body weight. Only sex was removed from the statistical model when the mediation analysis explored boys or girls separately. Sex, maturation, and body weight are relevant indicators influencing bone growth and lean body mass (24, 38, 39). Regarding maturation, we used the Peak Height Velocity (PHV), considered a somatic maturation indicator that subtracts the chronological age from Moore's prediction model (40). Adolescence is a crucial stage related to BMC. During this period, the peak bone mass is reached due to several bone turnover markers, which depend on age, gender, and maturation (41). Physical activity was estimated by the Chilean physical activity questionnaire validated in the population aged 8–13 years (42). Adolescents were asked about several physical activities, and a final score was estimated indicating their physical activity level. Physical activity is an important moderator linked to improvement of muscle mass and BMC in children and adolescents (43). For socioeconomic status, we used the School Vulnerability Index (SVI), which measures the vulnerability degree for pupils who attend public, subsidized, or private schools. The SVI includes the family socioeconomic status, parents/tutor educational level, students' health condition, physical and emotional wellbeing, and school location (44); hence, the SVI seems to be a strong predictor of socioeconomic status in Latin-American countries (45). The SVI scores range from 0 to 100 and are updated every year. Private schools are not considered in the SVI, as they do not receive contributions from the state, so they were assigned a value of 0. Thus, the schools were classified as low (<10), middle (≥10 to <60), and high (≥60) SVI.

### Statistical analysis

First, missing data were imputed based on the non-parametric missing value method using random forest through the “missForest” R package (46). Missing data ranges were between 1.2% (body weight) to 17.7% (neck circumference) and estimation error was 0.14% (numeric variables) and 0.23% (factor variables). Then, the Shapiro-Wilk test and Q-Q plots (quantile-quantile plot) were used to check normality data distribution. Non-normal distribution variables were normalized by the two-step method (47).

Secondly, a principal components analysis (PCA) was performed by a dimensionality-reduction method to establish a single fatness factor involving four indicators [two anthropometric factors: neck circumference (cm) and WHtR, and two BIA factors: total fat (kg) and visceral fat area (cm^2^)]. This PCA component was determined using the Varimax rotation, explaining 71.5% of the variance. The assumption check for Bartlett's Test of sphericity was significant (*p* < 0.001). Analyses were performed using JAMOVI based on the “psych” Package for R (48).

Third, a linear regression (B and 95% confidence interval [95%CI]) was performed to establish the interaction of fatness/ASMI by sex on BMC. Sex interaction was observed in both fatness and ASMI variables; thereby, three mediation models were run, only boys, only girls, and all together.

**Figure 1:**
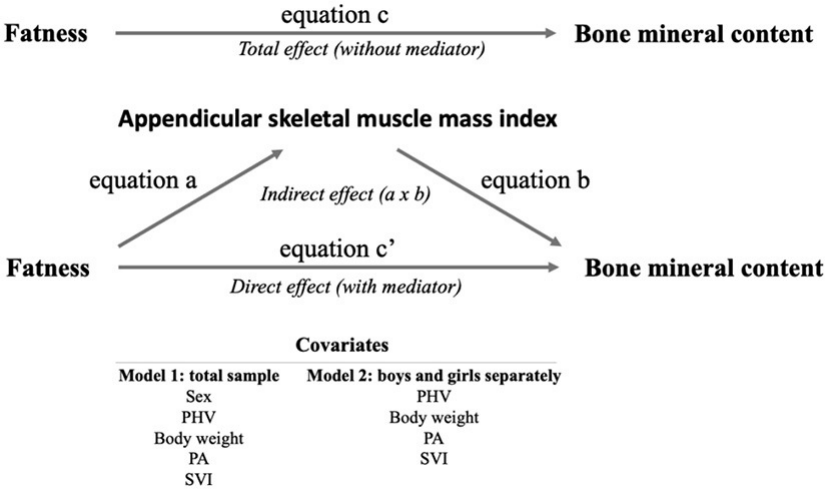
Mediation analysis approach. PHV, peak high velocity; PA, physical activity; SVI, school vulnerability index.

Finally, three mediation analyses were performed. The general mediation approach considered fatness as the predictor, ASMI as the mediator, and BMC as the outcome (Figure 1). Thus, mediation models were structured as follows: equation (a) consisted of the predictor by the mediator; equation (b) was defined as the mediator by the outcome; equation (c) consisted of the predictor by the outcome (Total effect); and finally, equation (c′) consisted of predictor and mediator by the outcome (Direct effect). The indirect effect was estimated by a^*^b equations, and it was significant if zero was outside the 95%CI (49, 50). Bootstrapping with 5,000 samples was performed (51). The percentage of mediation was estimated as 1 – (equation c′/equation c). Bias was reduced by adjusting analyses to relevant covariates such as sex, PHV, body weight, physical activity, and SVI. A *p* < 0.05 was set as the limit for statistical significance.

## Results

The descriptive characteristics of the adolescents are displayed in Table 1. Significant differences according to sex were found in almost all the variables analyzed. WHtR, neck circumference, BMC, muscle mass, physical activity, and ASMI were higher in boys, while PHV, fat mass, and visceral fat area were higher in girls. No differences were found only in height and BMI.

**Table 1:** Participant characteristics.

	**Total sample** ** (*n* = 1,296)**	**Boys** ** (*n* = 648)**	**Girls** ** (*n* = 648)**	***p***
Age (y)	11.9 ± 1.1	11.8 ± 1.2	11.9 ± 1.2	0.089
Weight (kg)	50.9 ± 12.0	50.1 ± 12.2	51.7 ± 11.7	0.013
Height (cm)	153.1 ± 9.3	153.0 ± 10.4	153.1 ± 8.1	0.929
BMI				
Normal BMI	737 (56.9%)	377 (58.2%)	360 (55.6%)	0.370
OW-OB	559 (43.1%)	271 (41.8%)	288 (44.4%)	
Peak height velocity (years)	−0.41 ± 1.3	−1.16 ± 1.0	0.34 ± 1.0	<0.01
Physical activity score	4.69 ± 1.4	4.91 ± 1.4	4.48 ± 1.3	<0.01
Waist-to-height ratio	0.46 ± 0.1	0.46 ± 0.1	0.45 ± 0.1	<0.01
Neck circumference (cm)	30.9 ± 2.4	31.4 ± 2.4	30.4 ± 2.3	<0.01
Bone mineral content (kg)	2.2 ± 0.5	2.3 ± 0.5	2.2 ± 0.4	<0.01
Fat mass (kg)	13.9 ± 7.4	12.4 ± 7.0	15.5 ± 7.5	<0.01
Muscle mass (kg)	19.8 ± 4.5	20.2 ± 5.0	19.4 ± 3.9	<0.01
Visceral fat area (cm^2^)	70.7 ± 33.2	67.4 ± 33.0	73.9 ± 33.1	<0.01
ASMI (kg/cm^2^)	8.4 ± 1.2	8.5 ± 1.2	8.2 ± 1.1	<0.01
School vulnerability index				
Low	326 (25.2%)	144 (22.0%)	182 (28.1%)	0.038
Middle	360 (27.8%)	181 (27.9%)	179 (27.6%)	
High	610 (47.1%)	323 (49.8%)	287 (44.3%)	

Values are mean ± standard deviation or frequency (%). BMI, Body Mass Index; OW-OB, overweight-obese; ASMI, Appendicular Skeletal Muscle Mass Index. The *p* < 0.05 indicates significant differences between boys and girls. Both t-tests and chi-square tests were performed to test for differences between groups.

**Figure 2:**
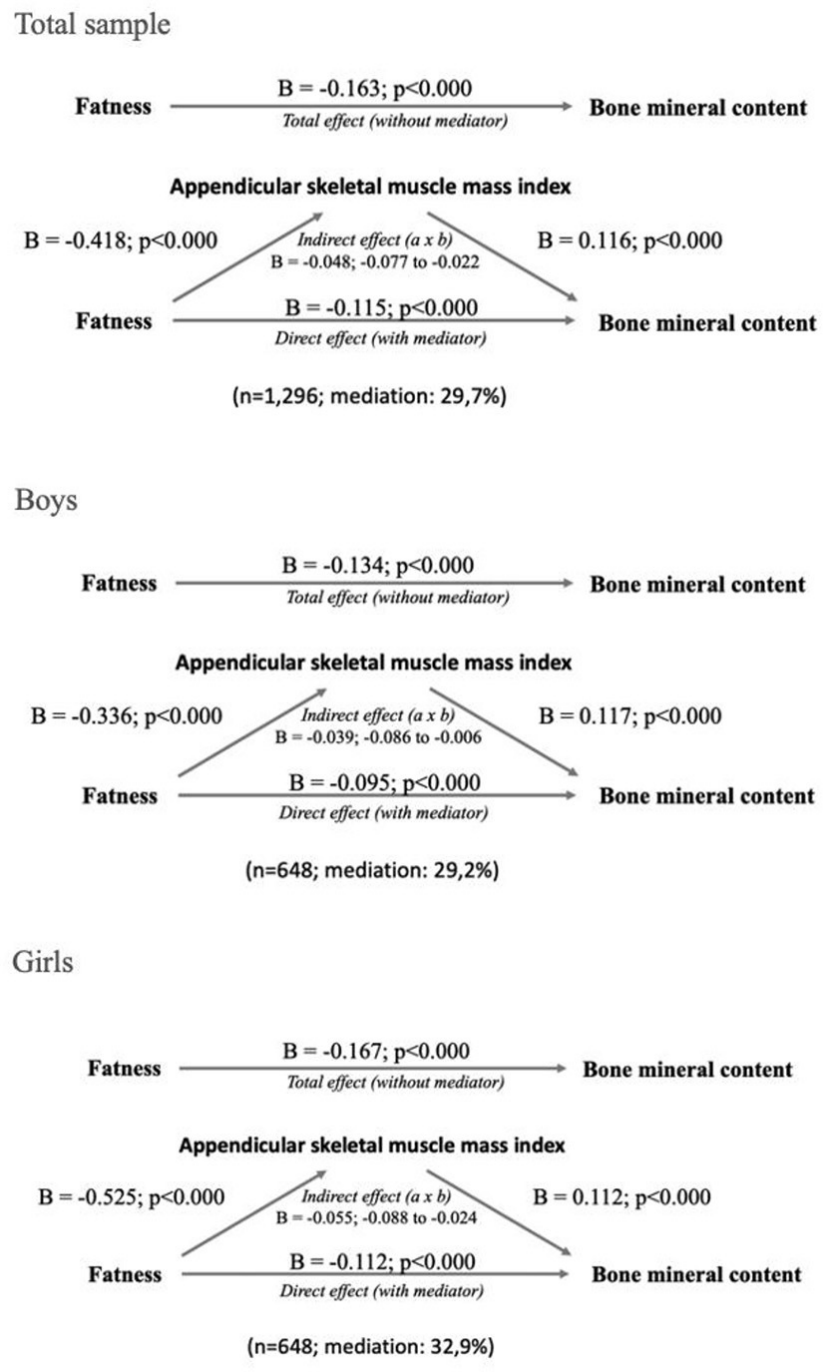
Total sample and sex-specific mediation analyses. Unstandardized beta values are presented.

Figure 2 presents the general and sex-specific mediation analyses. Overall, fatness was negatively associated with BMC in the total sample, boys, and girls, being higher than boys in the last group (B = −0.170 vs. B = −0.139; *p* < 0.001, respectively). Moreover, fatness was negatively associated with ASMI in the total sample, boys, and girls, being higher than boys in the last group (B = −0.528 vs. B = −0.353; *p* < 0.001, respectively). Otherwise, ASMI was positively related to BMC in the total sample, boys, and girls, being slightly higher in boys than girls (B = 0.118 vs. B = 0.106; *p* < 0.001, respectively). Regarding mediation analyses, total, direct, and indirect effects were statically significant, indicating a partial ASMI mediation in the inverse relationship between fatness and BMC. For the total sample and boys, the ASMI mediation role accounted for 29.9%, while girls accounted for 32.8%.

Besides, we explore the ASMI mediation effect in the total sample according to BMI status. Adolescents with normal-BMI (*n* = 737) presented a significant indirect effect, representing a partial mediation of 37.6% (B = −0.061, 95%CI −0.095 to −0.024), while the overweight-obese group (*n* = 559) also showed a significant indirect effect representing a partial mediation 23.9% (B= −0.040, 95%CI −0.096 to −0.033).

## Discussion

The present study aimed to establish the mediating role of the ASMI in the association between fatness (computed by four fatness indicators) with BMC in a large sample of Chilean adolescents. The main findings indicate that ASMI significantly mediates (around 30%) the inverse relationship between fatness and BMC, and this mediation seems to be slightly higher in girls than boys. Besides, a difference of 13.7% in ASMI's mediation effect was found among BMI groups, being higher in adolescents with normal nutritional status. To the best of our knowledge, this is the first study that analyzes the ASMI in the negative impact of adolescents' fatness on bone health.

Previous studies and reviews have addressed the close interaction between obesity, muscle mass, and bone metabolism (9, 16, 52, 53). At the cellular level, the muscle, bone, and fat cross-talk evidences that adipocyte cells release a series of adipokines (pro-inflammatory and immunomodulatory molecules) that up-regulate osteoclast formation and activation, lead to chronic low-grade inflammation and alter cellular protein metabolism promoting bone fragility and sarcopenia (9, 52–55). While at the behavioral level, fatness can lead to physical inactivity but not the other way around (56, 57). Thus, low physical activity levels reduce muscle mass and bone content, promoting adiposity stored in children and adolescents (17, 18) and reinforcing this vicious circle. Therefore, the balance between adipokines, myokines, and osteokines is essential to regulating bone health in this population (16) but especially during adolescence, where diet, physical activity, and the hormonal milieu play a decisive role (9).

In this regard, our main finding contributes to this research area by showing the significant but partial mediation role of a muscle mass indicator adjusted for height on the relationship between fatness and BMC. In children and adolescents, ASMI, and strength are strongly associated (58), and both have been associated with bone health in the transition from childhood to adulthood (48, 59). Thereby, physical activity plays a fundamental role in promoting healthy muscle mass in this population. Adolescents with a higher level of physical activity present better ASMI and lower body fat percentage than those with lower levels of physical activity (60). Hence, physical activity enhances the development, growth, and quality of bone tissue by biomechanical traction when the muscle is subjected to external loads such as resistance training reported in children, adolescents, and adults (61, 62). For instance, myostatin (i.e., myokine produced and released by myocytes that acts on muscle cells to inhibit muscle growth) is regulated by physical activity and, in both animal and human studies, reduces its expression and increases bone turnover, leading to an increase in total bone mass (63).

Paradoxically, it has been documented that increased body weight generates a “training effect” in the lower limb in obese people through adolescence to old age; however, their muscle quality seems reduced (64). Further, Han et al. (65) reported that adolescents with greater body mass index showed greater bone mineral density, but this relationship was significant just when they presented greater skeletal muscle mass, calcium supplement intake, and lower body fat mass. In fact, recent evidence indicated that fat mass did not predict adolescent bone mineral density (7). This evidence is consistent with our finding adjusted for adolescent body weight, and also with our results showing that the mediation effect of ASMI seems to be higher (13.7%) in adolescents with normal-BMI.

Important to note is that we found a slightly higher mediation role of ASMI in girls than boys. Our finding is supported by evidence showing that Indian adolescent girls have lower ASMI than boys (24); thus, improving muscle mass could be a relevant target in this population to mediate the inverse association between fatness and bone health. Additionally, a recent university study found that girls with low physical activity levels had significantly lower ASMI than boys, specifically in lower extremity muscles (66). This concern may be even more critical in overweight/obese girls because they displayed higher intramuscular fat (lower muscle density) and low lean mass than their normal-weight lean peers (67). Thereby, increasing ASMI through physical activity (i.e., resistance training) would help to increase bone quality through fat reduction (62, 68, 69). This suggestion has practical applicability, as a meta-analysis (70) that evaluated the effect of interventions with high-intensity physical exercise and sports mini-games at school showed that girls are more responsive to increasing bone mineral density than boys.

The plausible explanation for the mediating role of ASMI in the inverse relationship between fatness and BMC can be described by reviewing previous studies. For example, Liu et al. (71) found that normal-weight and obese women (over a wide age range) had similar bone mineral density at the spine and femoral neck. This finding implies that the weight/obesity relationship does not necessarily translate into better bone mineral density at different sites of the skeleton; however, lean body mass had a strong positive relationship with bone mineral density. Indeed, the authors showed that the positive association between overweight and bone mineral density is not constant, and it presents a limit (around 33% of fat mass).

At the physiological level, several mechanisms converge simultaneously, improving bone health markers. For instance, adolescents who participated in 1 year of interdisciplinary treatment (physical exercise, nutritional, psychological counseling, and others) showed enhanced body composition (reduced body fat mass and increased body lean mass and bone mineral content) (72). Besides, an improvement in inflammatory markers was observed, but a positive correlation was mainly found between the adiponectin/leptin ratio and osteocalcin. Interestingly, both lean and body fat mass were predictors of osteocalcin; thus, this study gives supports—to some extent—the mediator role of ASMI in the association between fatness and BMC in adolescents.

Finally, although ASMI showed a significant role in the relationship between fatness and BMC, its mediation was not complete. This means that fatness is a decisive factor that multidisciplinary strategies must address because enhancing muscle mass in adolescents does not seem to be the only way. In this sense, diet is vital because studies have shown that high consumption of ultra-processed products in adolescence can attenuate the beneficial effects of physical activity on skeletal muscle mass (17). Besides, dietary protein, vitamin D, and calcium modulate bone metabolism, and protein and vitamin D have been associated with higher lean mass, strength, and function (16). Thereby, future strategies to improve bone health in adolescents should also combine physical activity and diet.

## Strengths and limitations

On the one hand, this study presents some strengths worth noting. This study analyzed data from a large adolescent sample size, helping to bridge the gap in this research area. Besides, we compute a fatness factor by reducing the dimensionality of four well-known fatness indicators, increasing interpretability while minimizing information loss. Finally, to the best of our knowledge, this could be the first study to consider ASMI as a mediator factor between fatness and a relevant marker of bone health. On the other hand, several limitations should be considered when interpreting the results of this study. The cross-sectional design of this study makes it impossible to establish causality, thus the results should be taken with caution. A key concern is that the accuracy of BIA to measure some indicators can be underestimated/overestimated if the participants do not follow the standard protocol required for optimal and correct body composition analysis. Due to the context and school routine, we could not measure all adolescents at the same time of the day nor monitor their drinking and eating substantially, which could affect results. Nonetheless, we tried to reduce possible methodological biases as much as possible. Quality of diet and vitamin D were not considered as covariates.

## Conclusions

This study showed that fatness is an important indicator associated with lower BMC in adolescents; however, a higher ASMI level mediated around 30% of the inverse relationship between fatness and BMC. This mediation was slightly higher in girls than boys, and in normal-weigh adolescents. Thus, our results highlighted that promoting healthy habits (i.e., physical activity, physical fitness) to reduce fatness and improve muscle mass is crucial, especially in adolescence, to contribute to optimal bone health. Moreover, these findings are relevant in public health and primary care to encourage bone health in childhood, reducing the incidence of early osteopenia and osteoporosis.

## Data availability statement

The datasets presented in this article are not readily available because of ethical restrictions. Requests to access the datasets should be directed to carlos.cristi.montero@gmail.com.

## Ethics statement

The studies involving human participants were reviewed and approved by Bioethics and Biosafety Committee of the Ethics Committee of Pontificia Universidad Católica de Valparaíso (BIOEPUCV-H103–2016). Written informed consent to participate in this study was provided by the participants' legal guardian/next of kin.

## Author contributions

CC-M conceived and designed the data analysis and manuscript and responsible for coordinating the study and acquiring the data. CC-M and HP-J wrote the first manuscript version. All authors contributed significantly to editing the manuscript and agreed to the final version.

## Funding

CC-M received funding for the Cogni-Action Project from the National Commission for Scientific and Technological Research CONICYT/FONDECYT INICIACION 2016 Grant No.: 11160703 (Chile).

## Conflict of interest

The authors declare that the research was conducted in the absence of any commercial or financial relationships that could be construed as a potential conflict of interest.

## Publisher's note

All claims expressed in this article are solely those of the authors and do not necessarily represent those of their affiliated organizations, or those of the publisher, the editors and the reviewers. Any product that may be evaluated in this article, or claim that may be made by its manufacturer, is not guaranteed or endorsed by the publisher.

## References

[1] Fan H Zhang X. Recent trends in overweight and obesity in adolescents aged 12 to 15 years across 21 countries. Pediatr Obes. (2022) 17:12839. 10.1111/IJPO.12839 34355535

[2] Obesity overweight. Available online at: https://www.who.int/news-room/fact-sheets/detail/obesity-and-overweight (accessed July 8, 2022).

[3] Chaput JP Willumsen J Bull F Chou R Ekelund U Firth J. 2020 WHO guidelines on physical activity and sedentary behaviour for children and adolescents aged 5–17 years: summary of the evidence. Int J Behav Nutr Phys Act. (2020) 17:1–9. 10.1186/S12966-020-01037-Z/TABLES/6 33239009 PMC7691077

[4] Baxter-Jones ADG Faulkner RA Forwood MR Mirwald RL Bailey DA. Bone mineral accrual from 8 to 30 years of age: an estimation of peak bone mass. J Bone Miner Res. (2011) 26:1729–39. 10.1002/JBMR.412 21520276

[5] Ambrosio MR Aliberti L Gagliardi I Franceschetti P Zatelli MC. Bone health in adolescence. Minerva Obstet Gynecol. (2021) 73:662–77. 10.23736/S2724-606X.20.04713-9 34905874

[6] Hui SL Slemenda CW Johnston CC. The contribution of bone loss to postmenopausal osteoporosis. Osteoporos Int. (1990) 1:30–4. 10.1007/BF01880413 2133638

[7] Pelegrini A Bim MA Alves AD Scarabelot KS Claumann GS Fernandes RA. Relationship between muscle strength, body composition and bone mineral density in adolescents. J Clin Densitom. (2022) 25:54–60. 10.1016/J.JOCD.2021.09.001 34756705

[8] Lopes KG Rodrigues EL Lopes MR da S Nascimento VA Pott A Guimarães R de CA. Adiposity metabolic consequences for adolescent bone health. Nutrients. (2022) 14:3260. 10.3390/NU14163260 38068812 PMC10708121

[9] Fintini D Cianfarani S Cofini M Andreoletti A Ubertini GM Cappa M. The bones of children with obesity. Front Endocrinol (Lausanne). (2020) 11:200. 10.3389/FENDO.2020.00200/BIBTEX 32390939 PMC7193990

[10] da Silva SV Renovato-Martins M Ribeiro-Pereira C Citelli M Barja-Fidalgo C. Obesity modifies bone marrow microenvironment and directs bone marrow mesenchymal cells to adipogenesis. Obesity (Silver Spring). (2016) 24:2522–32. 10.1002/OBY.21660 27753270

[11] Roy B Curtis ME Fears LS Nahashon SN Fentress HM. Molecular mechanisms of obesity-induced osteoporosis and muscle atrophy. Front Physiol. (2016) 7:439. 10.3389/FPHYS.2016.00439/BIBTEX 27746742 PMC5040721

[12] Lee WC Guntur AR Long F Rosen CJ. Energy metabolism of the osteoblast: implications for osteoporosis. Endocr Rev. (2017) 38:255. 10.1210/ER.2017-00064 28472361 PMC5460680

[13] Kessler J Koebnick C Smith N Adams A. Childhood obesity is associated with increased risk of most lower extremity fractures. Clin Orthop Relat Res. (2013) 471:1199–207. 10.1007/S11999-012-2621-Z 23054515 PMC3586019

[14] Saurabh K Hemang. Vitamin D status among overweight and obese adolescents. Biomed Biotechnol Res J. (2020) 4:162. 10.4103/BBRJ.BBRJ_11_20

[15] Pagnotti GM Styner M Uzer G Patel VS Wright LE Ness KK. Combating osteoporosis and obesity with exercise: leveraging cell mechanosensitivity. Nat Rev Endocrinol. (2019) 15:339–55. 10.1038/s41574-019-0170-1 30814687 PMC6520125

[16] Kirk B Feehan J Lombardi G Duque G. Muscle, bone, and fat crosstalk: the biological role of myokines, osteokines, and adipokines. Curr Osteoporos Rep. (2020) 18:388–400. 10.1007/S11914-020-00599-Y 32529456

[17] Hao G Pollock NK Harris RA Gutin B Su S Wang X. Associations between muscle mass, physical activity and dietary behaviour in adolescents. Pediatr Obes. (2019) 14:12471. 10.1111/IJPO.12471 30280506

[18] Nguyen VH. School-based exercise interventions effectively increase bone mineralization in children and adolescents. Osteoporos Sarcopenia. (2018) 4:39–46. 10.1016/J.AFOS.2018.05.002 30775541 PMC6362970

[19] Levine M. Assessing bone health in children and adolescents. Indian J Endocrinol Metab. (2012) 16:205. 10.4103/2230-8210.104040 23565379 PMC3603027

[20] Hou Y Xie Z Zhao X Yuan Y Dou P Wang Z. Appendicular skeletal muscle mass: a more sensitive biomarker of disease severity than BMI in adults with mitochondrial diseases. PLoS ONE. (2019) 14:e0219628. 10.1371/JOURNAL.PONE.0219628 31344055 PMC6657836

[21] Wells JCK. Toward body composition reference data for infants, children, and adolescents. Adv Nutr. (2014) 5:320S−9S. 10.3945/AN.113.005371 24829484 PMC4013189

[22] Blain H Jaussent A Thomas E Micallef JP Dupuy AM Bernard PL. Appendicular skeletal muscle mass is the strongest independent factor associated with femoral neck bone mineral density in adult and older men. Exp Gerontol. (2010) 45:679–84. 10.1016/J.EXGER.2010.04.006 20433916

[23] da Cruz GF Lunz TM de Jesus TR Costa MB Vidigal CV Albergaria BH. Influence of the appendicular skeletal muscle mass index on the bone mineral density of postmenopausal women. BMC Musculoskelet Disord. (2021) 22:861. 10.1186/S12891-021-04748-X 34627216 PMC8501937

[24] Marwaha RK Garg MK Bhadra K Mahalle N Mithal A Tandon N. Lean body mass and bone health in urban adolescents from northern India. Indian Pediatr. (2017) 54:193–8. 10.1007/S13312-017-1029-Y 28159946

[25] Kaster T Dooley FL Fitzgerald JS Walch TJ Annandale M Ferrar K. Temporal trends in the sit-ups performance of 9,939,289 children and adolescents between 1964 and 2017. J Sports Sci. (2020) 38:1913–23. 10.1080/02640414.2020.1763764 32567491

[26] Tomkinson GR Kaster T Dooley FL Fitzgerald JS Annandale M Ferrar K. Temporal trends in the standing broad jump performance of 10,940,801 children and adolescents between 1960 and 2017. Sport Med. (2020) 51:531–548. 10.1007/S40279-020-01394-6 33368030

[27] Orsso CE Tibaes JRB Oliveira CLP Rubin DA Field CJ Heymsfield SB. Low muscle mass and strength in pediatrics patients: why should we care? Clin Nutr. (2019) 38:2002–15. 10.1016/J.CLNU.2019.04.012 31031136

[28] Pisani P Renna MD Conversano F Casciaro E Di Paola M Quarta E. Major osteoporotic fragility fractures: risk factor updates and societal impact. World J Orthop. (2016) 7:171–81. 10.5312/WJO.V7.I3.171 27004165 PMC4794536

[29] Solis-Urra P Olivares-Arancibia J Suarez-Cadenas E Sanchez-Martinez J Rodríguez-Rodríguez F Ortega FB. Study protocol and rationale of the “cogni-action project” a cross-sectional and randomized controlled trial about physical activity, brain health, cognition, and educational achievement in schoolchildren. BMC Pediatr. (2019) 19:1–16. 10.1186/s12887-019-1639-8 31349791 PMC6659252

[30] von Elm E Altman DG Egger M Pocock SJ Gøtzsche PC Vandenbroucke JP. The strengthening the reporting of observational studies in epidemiology (STROBE) statement: guidelines for reporting observational studies. Int J Surg. (2014) 12:1495–9. 10.1016/J.IJSU.2014.07.013 25046131

[31] Cole TJ Lobstein T. Extended international (IOTF) body mass index cut-offs for thinness, overweight and obesity. Pediatr Obes. (2012) 7:284–94. 10.1111/J.2047-6310.2012.00064.X 22715120

[32] Mucelin E Traebert J Zaidan MA Piovezan AP Nunes RD Traebert E. Accuracy of neck circumference for diagnosing overweight in six- and seven-year-old children. J Pediatr (Rio J). (2021) 97:559–63. 10.1016/J.JPED.2020.11.005 33358966 PMC9432317

[33] Sehn AP Brand C Welser L Gaya AR Agostinis-Sobrinho C Cristi-Montero C. Neck circumference and cardiometabolic risk in children and adolescents: the moderator role of cardiorespiratory fitness. BMC Pediatr. (2021) 21:234. 10.1186/S12887-021-02696-Y 34001053 PMC8127299

[34] Juonala M Magnussen CG Berenson GS Venn A Burns TL Sabin MA. Childhood adiposity, adult adiposity, and cardiovascular risk factors. N Engl J Med. (2011) 365:1876–85. 10.1056/NEJMOA1010112 22087679

[35] Arnaiz P Grob F Cavada G Domínguez A Bancalari R Cerda V. La razón cintura estatura en escolares no varía con el género, la edad ni la maduración puberal. Rev Med Chil. (2014) 142:574–8. 10.4067/S0034-98872014000500004 25427013

[36] Ng BK Liu YE Wang W Kelly TL Wilson KE Schoeller DA. Validation of rapid 4-component body composition assessment with the use of dual-energy X-ray absorptiometry and bioelectrical impedance analysis. Am J Clin Nutr. (2018) 108:708–15. 10.1093/AJCN/NQY158 30099474 PMC7263310

[37] Jayanama K Putadechakun S Srisuwarn P Vallibhakara SAO Chattranukulchai Shantavasinkul P Sritara C. Evaluation of body composition in hemodialysis thai patients: comparison between two models of bioelectrical impedance analyzer and dual-energy X-Ray absorptiometry. J Nutr Metab. (2018) 2018:4537623. 10.1155/2018/4537623 30174950 PMC6098916

[38] Leonard MB Elmi A Mostoufi-Moab S Shults J Burnham JM Thayu M. Effects of sex, race, and puberty on cortical bone and the functional muscle bone unit in children, adolescents, and young adults. J Clin Endocrinol Metab. (2010) 95:1681. 10.1210/JC.2009-1913 20157194 PMC2853999

[39] Wang L Xu Z Li N Meng X Wang S Yu C. The association between overweight and obesity on bone mineral density in 12 to 15 years old adolescents in China. Medicine (Baltimore). (2021) 100:e26872. 10.1097/MD.0000000000026872 34397903 PMC8360441

[40] Moore SA McKay HA Macdonald H Nettlefold L Baxter-Jones ADG Cameron N. Enhancing a somatic maturity prediction model. Med Sci Sports Exerc. (2015) 47:1755–64. 10.1249/MSS.0000000000000588 25423445

[41] Cao B Liu M Luo Q Wang Q Liu M Liang X. The Effect of BMI, Age, gender, and pubertal stage on bone turnover markers in Chinese children and adolescents. Front Endocrinol (Lausanne). (2022) 13:977. 10.3389/FENDO.2022.880418/BIBTEX 35769079 PMC9234688

[42] Claude Godard M María Del Pilar Rodríguez N Díaz N Lydia Lera M Gabriela Salazar R Raquel Burrows A. Valor de un test clínico para evaluar actividad física en niños. Rev Med Chil. (2008) 136:1155–62. 10.4067/S0034-98872008000900010 19030660

[43] Pimentel DV Suttkus A Vogel M Lacher M Jurkutat A Poulain T. Effect of physical activity and BMI SDS on bone metabolism in children and adolescents. Bone. (2021) 153:116131. 10.1016/J.BONE.2021.116131 34314901

[44] López V Oyanedel JC Bilbao M Torres J Oyarzún D Morales M. School achievement and performance in Chilean high schools: the mediating role of subjective wellbeing in school-related evaluations. Front Psychol. (2017) 8:1189. 10.3389/FPSYG.2017.01189/BIBTEX 28769838 PMC5509788

[45] Flores-Mendoza C Rosas R Gallegos M Ardila R Lucio ME Colareta NR. Intelligence measurement and school performance in Latin America: A report of the study of Latin American intelligence project. Intell Meas Sch Perform Lat Am A Rep Study Lat Am Intell Proj. (2018) 2018:1–124. 10.1007/978-3-319-89975-6/COVER

[46] Stekhoven DJ Bühlmann P. MissForest—non-parametric missing value imputation for mixed-type data. Bioinformatics. (2012) 28:112–8. 10.1093/BIOINFORMATICS/BTR597 22039212

[47] Templeton GF. A two-step approach for transforming continuous variables to normal: implications and recommendations for IS research. Commun Assoc Inf Syst. (2011) 28:4. 10.17705/1CAIS.02804

[48] Revelle WR. psych: Procedures for Personality and Psychological Research. Uniw ślaski. (2017) 343–354. 10.2/JQUERY.MIN.JS20392272

[49] Field A. Discovering Statistics Using IBM SPSS Statistics And Sex and Drugs and Rock “N” Roll, 4th Edition. Sage, Los Angeles, London, New Delhi: References - Scientific Research Publishing (2013). Available online at: https://www.scirp.org/(S(351jmbntvnsjt1aadkposzje))/reference/ReferencesPapers.aspx?ReferenceID=2046660 (accessed August 15, 2022).

[50] Hayes AF Rockwood NJ. Regression-based statistical mediation and moderation analysis in clinical research: observations, recommendations, and implementation. Behav Res Ther. (2017) 98:39–57. 10.1016/J.BRAT.2016.11.001 27865431

[51] Preacher KJ Hayes AF. Asymptotic and resampling strategies for assessing and comparing indirect effects in multiple mediator models. Behav Res Methods. (2008) 40:879–891. 10.3758/BRM.40.3.879 18697684

[52] Greco EA Lenzi A Migliaccio S. The obesity of bone. Ther Adv Endocrinol Metab. (2015) 6:273. 10.1177/2042018815611004 26623005 PMC4647134

[53] López-Gómez JJ Pérez Castrillón JL de Luis Román DA. Impact of obesity on bone metabolism. Endocrinol Nutr (English Ed). (2016) 63:551–9. 10.1016/J.ENDOEN.2016.08.013 27744014

[54] Festa A D'Agostino R Williams K Karter AJ Mayer-Davis EJ Tracy RP. The relation of body fat mass and distribution to markers of chronic inflammation. Int J Obes Relat Metab Disord. (2001) 25:1407–15. 10.1038/SJ.IJO.0801792 11673759

[55] Xia Z Cholewa J Zhao Y Shang HY Yang YQ Pessôa KA. Targeting inflammation and downstream protein metabolism in sarcopenia: a brief up-dated description of concurrent exercise and leucine-based multimodal intervention. Front Physiol. (2017) 8:434. 10.3389/FPHYS.2017.00434 28690550 PMC5479895

[56] Metcalf BS Hosking J Jeffery AN Voss LD Henley W Wilkin TJ. Fatness leads to inactivity, but inactivity does not lead to fatness: a longitudinal study in children (EarlyBird 45). Arch Dis Child. (2011) 96:942–7. 10.1136/ADC.2009.175927 20573741

[57] Hjorth MF Chaput JP Ritz C Dalskov SM Andersen R Astrup A. Fatness predicts decreased physical activity and increased sedentary time, but not vice versa: support from a longitudinal study in 8- to 11-year-old children. Int J Obes. (2014) 38:959–65. 10.1038/IJO.2013.229 24304596

[58] Marriott P Confortin SC Yanet Gómez Aristizábal L Luanna M Martins Bragança B Cavalcante LC. Are fat mass and lean mass associated with grip strength in adolescents? Nutrients. (2022) 14:3259. 10.3390/NU14163259 36014765 PMC9416332

[59] Deng KL Yang WY Hou JL Li H Feng H Xiao SM. Association between body composition and bone mineral density in children and adolescents: a systematic review and meta-analysis. Int J Environ Res Public Health. (2021) 18:12126. 10.3390/IJERPH182212126 34831882 PMC8618958

[60] Ito T Sugiura H Ito Y Noritake K Ochi N. Relationship between the skeletal muscle mass index and physical activity of Japanese children: a cross-sectional, observational study. PLoS ONE. (2021) 16:251025. 10.1371/JOURNAL.PONE.0251025 34038448 PMC8153420

[61] Specker B Thiex NW Sudhagoni RG. Does exercise influence pediatric bone? A systematic review. Clin Orthop Relat Res. (2015) 473:3658. 10.1007/S11999-015-4467-7 26208606 PMC4586223

[62] Gómez AL Kraemer WJ Maresh CM Lee EC Szivak TK Caldwell LK. Resistance training and milk-substitution enhance body composition and bone health in adolescent girls. J Am Coll Nutr. (2021) 40:193–210. 10.1080/07315724.2020.1770636 32521207

[63] Guo B Zhang ZK Liang C Li J Liu J Lu A. Molecular communication from skeletal muscle to bone: a review for muscle-derived myokines regulating bone metabolism. Calcif Tissue Int. (2017) 100:184–92. 10.1007/S00223-016-0209-4/FIGURES/1 27830278

[64] Tomlinson DJ Erskine RM Morse CI Winwood K Onambélé-Pearson G. The impact of obesity on skeletal muscle strength and structure through adolescence to old age. Biogerontology. (2016) 17:467. 10.1007/S10522-015-9626-4 26667010 PMC4889641

[65] Han C Kim H Kim S. Effects of adolescents' lifestyle habits and body composition on bone mineral density. Int J Environ Res Public Health. (2021) 18:6170. 10.3390/IJERPH18116170 34200352 PMC8201294

[66] Oshita K Myotsuzono R. An association between the physical activity level and skeletal muscle mass index in female university students with a past exercise habituation. Osteoporos Sarcopenia. (2021) 7:146–52. 10.1016/J.AFOS.2021.10.002 35005251 PMC8714470

[67] Cheng S Wiklund P. The effects of muscle mass and muscle quality on cardio-metabolic risk in peripubertal girls: a longitudinal study from childhood to early adulthood. Int J Obes (Lond). (2018) 42:648–54. 10.1038/IJO.2017.267 29081501

[68] DuBose KD Eisenmann JC Donnelly JE. Aerobic fitness attenuates the metabolic syndrome score in normal-weight, at-risk-for-overweight, and overweight children. Pediatrics. (2007) 120:e1262–8. 10.1542/PEDS.2007-0443 17974719

[69] Vandoni M Calcaterra V Pellino VC De Silvestri A Marin L Zuccotti GV. “Fitness and Fatness” in children and adolescents: an Italian cross-sectional study. Children. (2021) 8:762. 10.3390/CHILDREN8090762 34572192 PMC8470229

[70] Mello JB Pedretti A García-Hermoso A Martins CML Gaya AR Duncan MJ. Exercise in school physical education increase bone mineral content and density: systematic review and meta-analysis. Eur J Sport Sci. (2021) 22:1618–29. 10.1080/17461391.2021.1960426 34328066

[71] Liu PY Ilich JZ Brummel-Smith K Ghosh S. New insight into fat, muscle and bone relationship in women: determining the threshold at which body fat assumes negative relationship with bone mineral density. Int J Prev Med. (2014) 5:1452. 25538842 PMC4274553

[72] Campos RM da S Masquio DCL Corgosinho FC de Carvalho-Ferreira JP Netto BDM Clemente APG. Relationship between adiponectin and leptin on osteocalcin in obese adolescents during weight loss therapy. Arch Endocrinol Metab. (2018) 62:275–84. 10.20945/2359-3997000000039 29791651 PMC10118784

